# Fast 2D/3D object representation with growing neural gas

**DOI:** 10.1007/s00521-016-2579-y

**Published:** 2016-09-22

**Authors:** Anastassia Angelopoulou, Jose Garcia Rodriguez, Sergio Orts-Escolano, Gaurav Gupta, Alexandra Psarrou

**Affiliations:** 1 Faculty of Science and Technology, University of Westminster, 115 New Cavendish Street, Middlesex, W1W 6UW UK; 2 Department of Computing Technology, University of Alicante, PO Box 99, 03080 Alicante, Spain; 3 Faculty of Media, Arts and Design, University of Westminster, Northwick Park, Middlesex, HA1 3TP UK

**Keywords:** Minimum description length, Self-organising networks, Shape modelling, Clustering

## Abstract

This work presents the design of a real-time system to model visual objects with the use of self-organising networks. The architecture of the system addresses multiple computer vision tasks such as image segmentation, optimal parameter estimation and object representation. We first develop a framework for building non-rigid shapes using the growth mechanism of the self-organising maps, and then we define an optimal number of nodes without overfitting or underfitting the network based on the knowledge obtained from information-theoretic considerations. We present experimental results for hands and faces, and we quantitatively evaluate the matching capabilities of the proposed method with the topographic product. The proposed method is easily extensible to 3D objects, as it offers similar features for efficient mesh reconstruction.

## Introduction

The images captured of hand gestures, which are effectively a 2D projection of a 3D object, can become very complex for any recognition system. Systems that follow a model-based method [1, 32] require an accurate 3D model that captures efficiently the hand’s high Degrees of Freedom (DOF) articulation and elasticity. The main drawback of this method is that it requires massive calculations which makes it unrealistic for real-time implementation. Since this method is too complicated to implement, the most widespread alternative is the feature-based method [16] where features such as the geometric properties of the hand can be analysed using either Neural Networks (NNs) [34, 36] or stochastic models such as Hidden Markov Models (HMMs) [6, 35].

However, for the accurate analysis of the hand’s properties, a suitable segmentation that separates the object of interest from the background is needed. Segmentation is a pre-processing step in many computer vision applications. These applications include visual surveillance [5, 10, 18, 20], and object tracking [15, 17, 26]. While a lot of research has been focused on efficient detectors and classifiers, little attention has been paid to efficiently labelling and acquiring suitable training data. Existing approaches to minimise the labelling effort [19, 21, 24, 30] use a classifier which is trained in a small number of examples. Then the classifier is applied on a training sequence, and the detected patches are added to the previous set of examples. Levin et al. [21] start with a small set of hand labelled data and generate additional labelled examples by applying co-training of two classifiers. Nair and Clark [24] use motion detection to obtain the initial training set. Lee et al. [21] use a variant of eigentracking to obtain the training sequence for face recognition and tracking. Sivic et al. [30] use boosting orientation-based features to obtain training samples for their face detector. A disadvantage of these approaches is that either a manual initialization [19] or a pre-trained classifier is needed to initialise the learning process. Having a sequence of images, this can be avoided by using an incremental model.

We decided to use NNs to represent the geometric properties of objects, and more specifically the self-organising maps (SOMs), due to their incremental nature. One of these SOM-based methods is the growing cell structures (GCS) algorithm [8], which is a model formed incrementally. However, it constrains the connections between the nodes, so any model produced during the training stage is always topologically equivalent to the initial topology. The Topology Representing Networks (TRN) approach, proposed by Martinez and Schulten [22], does not have a fixed structure and also does not impose any constraint on the connection between the nodes. In contrast, this network has a pre-established number of nodes and, therefore, it is not able to generate models with different resolutions. The algorithm was also coined with the term Neural Gas (NG) due to the dynamics of the feature vectors during the adaptation process, which distribute themselves like a gas within the data space. However, as the NG has a fixed number of nodes, it is necessary to have some a priori information about the input space to pre-establish the size of the network. This model was extended by Fritzke [9] proposing the Growing Neural Gas (GNG) network, which combined the flexible structure of the NG with a growing strategy. Moreover, the learning adaptation step was slightly modified. This extension enabled the neural network to use the already detected topological information while training in order to conform to the geometry. This approach has the capability to add neurons while preserving the topology of the input space.

Although the use of the SOM-based techniques of NG, GCS or GNG for various data inputs has already been studied and successful results have been reported [4, 13, 14, 27, 31, 32], there are some limitations that still persist. Most of these works assumed noise-free environments and low complexity distributions. Therefore, applying these methods on challenging real world data obtained using noisy 2D^1^ and 3D^2^ sensors is our main study. These particular non-invasive sensors have been used in the associated experiments and are typical, contemporary technology.

In this work, we extend the method presented in [2] for object representation using the GNG algorithm. This work extends the already proposed method by considering elimination of noisy connections during the learning process and by applying it to 3D datasets. The method is used for the representation of two-dimensional outline of hands and ventricles, which is extended to 3D. Furthermore, we are interested in the minimisation of the user intervention in the learning process; thus, we utilise an automatic criterion for maximum node growth based on topological parameters. We achieve that by taking into consideration that human skin has a relatively unique colour and the complexity or simplicity of the proposed model is decided by information-theoretic measures.

The remainder of the paper is organised as follows. Section 2 introduces the framework for object modelling using topological relations. Section 3 proposes an approach to minimise the user intervention in the termination of the network using knowledge obtained from information-theoretic considerations. In Sect. 4 a set of experimental results is presented that includes 2D and 3D representations before conclusions are drawn in Sect. 5.

## Characterising 2D objects with modified GNG

GNG [9] is an unsupervised incremental self-organising network independent of the topology of the input distribution or space. It uses a growth mechanism inherited from the Growth Cell Structure [8] together with the Competitive Hebbian Learning (CHL) rule [22] to construct a network of the input date set. In the GNG algorithm [9], the growing process starts with two nodes, and new nodes are incrementally inserted until a predefined conditioned is satisfied, such as the maximum number of nodes or available time. During the learning process, local error measures are gathered to determine where to insert new nodes. New nodes are inserted near the node with the highest accumulated error and new connections between the winner node and its topological neighbours are created.

Identifying the points of the image that belong to objects allows the GNG network to obtain an induced Delaunay triangulation of the objects. In other words, to obtain an approximation of the geometric appearance of the object. Let an object $\mathbf {\textit{O}} = [\mathbf {\textit{O}}_{G}, \mathbf {\textit{O}}_{A}]$ be defined by its geometry and its appearance. The geometry provides a mathematical description of the object’s shape, size, and parameters such as translation, rotation, and scale. The appearance defines a set of the object’s characteristics such as colour, texture, and other attributes.

Given a domain $\mathbf {S}\subseteq \mathbb {R}^2$, an image intensity function $\mathbf {I}(x,y)\in \mathbb {R}$ such that $\mathbf {I} : \mathbf {S} \rightarrow [0, \mathbf {I}_{\max }]$, and an object $\mathbf {\textit{O}}$, its standard potential field $\varPsi _{T} (x,y) = f_{T}(I(x,y))$ is the transformation $\varPsi _{T}: \mathbf {S} \rightarrow [0, 1]$ which associates with each point $(x,y)\in \mathbf {S}$ the degree of compliance with the visual property *T* of the object $\mathbf {\textit{O}}$ by its associated intensity $\mathbf {I}(x,y)$.

Considering:

- The input distribution as the set of points in the image:
  1 $${\mathbf {A}}= {\mathbf {S}}$$
  2 $$\xi _{w}= (x,y)\in {\mathbf {S}}$$
- The probability density function according to the standard potential field obtained for each point of the image:
  3 $$p(\xi _{w}) = p(x,y) = \varPsi _{T} (x,y)$$

Learning takes place with our modified GNG algorithm where wrong edges in the network are eliminated and the final graph is normalised. Algorithms 1 and 2 describe our extended GNG. During this process, the neural network is obtained which preserves the topology of the object ${\mathbf {\textit{O}}}$ from a certain feature *T*. Therefore, from the visual appearance ${\textit{O}}_{A}$ of the object is obtained an approximation to its geometric appearance $\mathbf {\textit{O}}_{G}$. Henceforth, the Topology Preserving Graph $TPG = \langle A,C\rangle$ is defined with a set of vertices (nodes) *A* and a set of connections (edges) *C*. To speed up the learning, we used the faster Manhattan distance [23] compared to the Euclidean distance in the original algorithm [9].

**Fig. 1:**
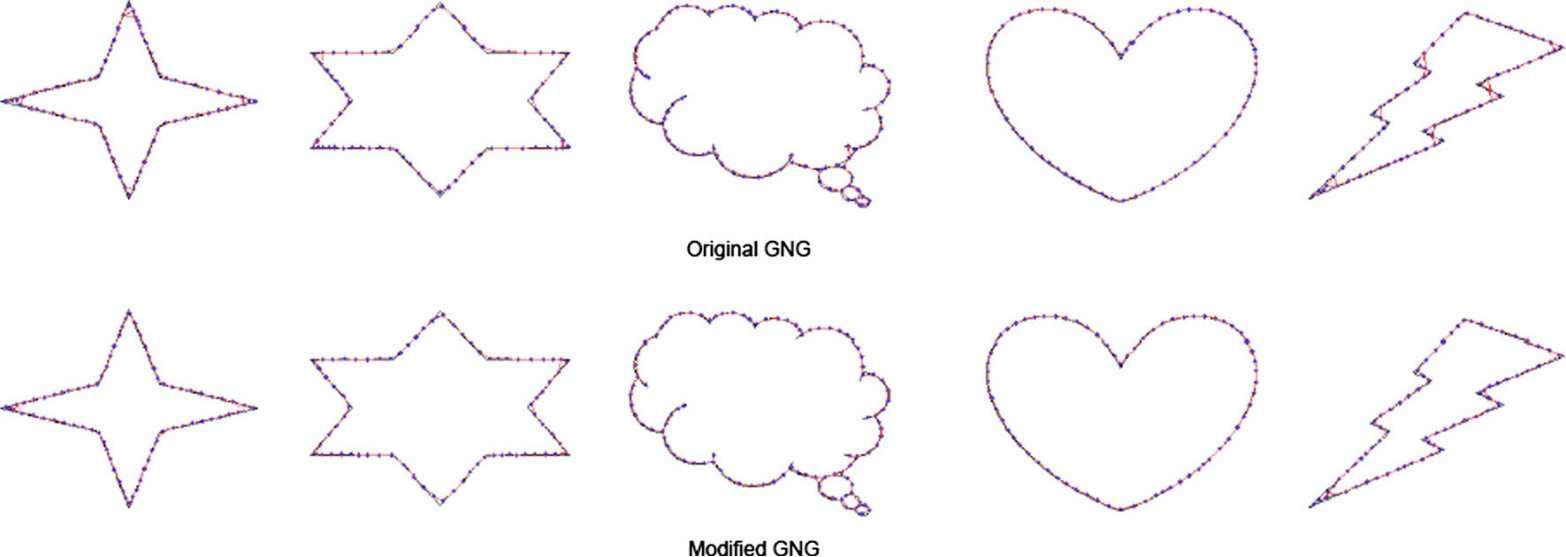
The *first row* shows the original GNG while the *second row* shows the modified GNG. With the modified GNG any wrong corrections to corners and curvatures have been eliminated

Figure 1 compares the original GNG algorithm with the modified GNG in 5 simple shapes with curvatures and corners.

We test the performance of the modified GNG by quantitative measures as shown in Table 1. The two measures of topological correctness that we used are the mean Quantisation Error (qe) and the Topology Preservation Error (te) [33], shown in Eqs. 4 and 5 respectively. There are *N* pixels, or reference vectors $\overrightarrow{x_{c}}$, representing the input space in the GNG network. Each node $c\in N$ has its associated reference vector $\{x_{c}\}_{c=1}^{N}\in \mathbb {R}^{q}$. The reference vectors indicate the nodes’ position or *receptive field centre* in the input distribution. We first analyse the quantisation error for each node with the Euclidean distance to its Best Matching Unit (BMU)$_{m_{\overrightarrow{x_{c}}}}$. The Best Matching Unit (BMU)$_{m_{\overrightarrow{x_{c}}}}$ is the node whose reference vector is closest to the input signal $(\xi _{w})$. The mean quantization error (qe) is the average distance between each reference vector and its BMU. For the calculation of topographic error, there is a function $u(\overrightarrow{x_{c}})$ that is 1 if $\overrightarrow{x_{c}}$ data vectors first and second BMUs are adjacent and 0 otherwise. The modified version of GNG produces a significant speed increase, with better connections in corners and angles and better topology preservation (less error).

4 $$\begin{aligned} \hbox{qe} & = \frac{1}{N} \sum _{c=1}^{N} \parallel \overrightarrow{x_{c}} - m_{\overrightarrow{x_{c}}}\parallel \end{aligned}$$

5 $$\begin{aligned} \hbox{te} & = \frac{1}{N} \sum _{c=1}^{N} u(\overrightarrow{x_{c}}) \end{aligned}$$

**Table 1:** Topology Preservation measures of the original vs. modified GNG with respect to frames per second (fps)

Shape	Nodes	Original GNG			Modified GNG		
		Fps	QE	TE	Fps	QE	TE
Star-4	71	1.16	2.7551	0	7.30	2.6375	0
Star-6	74	1.11	2.9564	0	6.06	2.9073	0.0014
Cloud	97	0.61	2.7275	0	5.26	2.6561	0
Heart	70	1.38	2.9337	0	5.24	2.9347	0
Lightning	71	1.04	2.9391	0	6.99	2.8138	0

As reflected in Table 1, GNG modified version provided lower quantization and topology preservation errors due to the deletion of wrong edges for most cases. However, in a few cases, wrong edges provide a shorter distance between input space and the Delaunay triangulation obtained (see Star-6 TE).

**Fig. 2:**
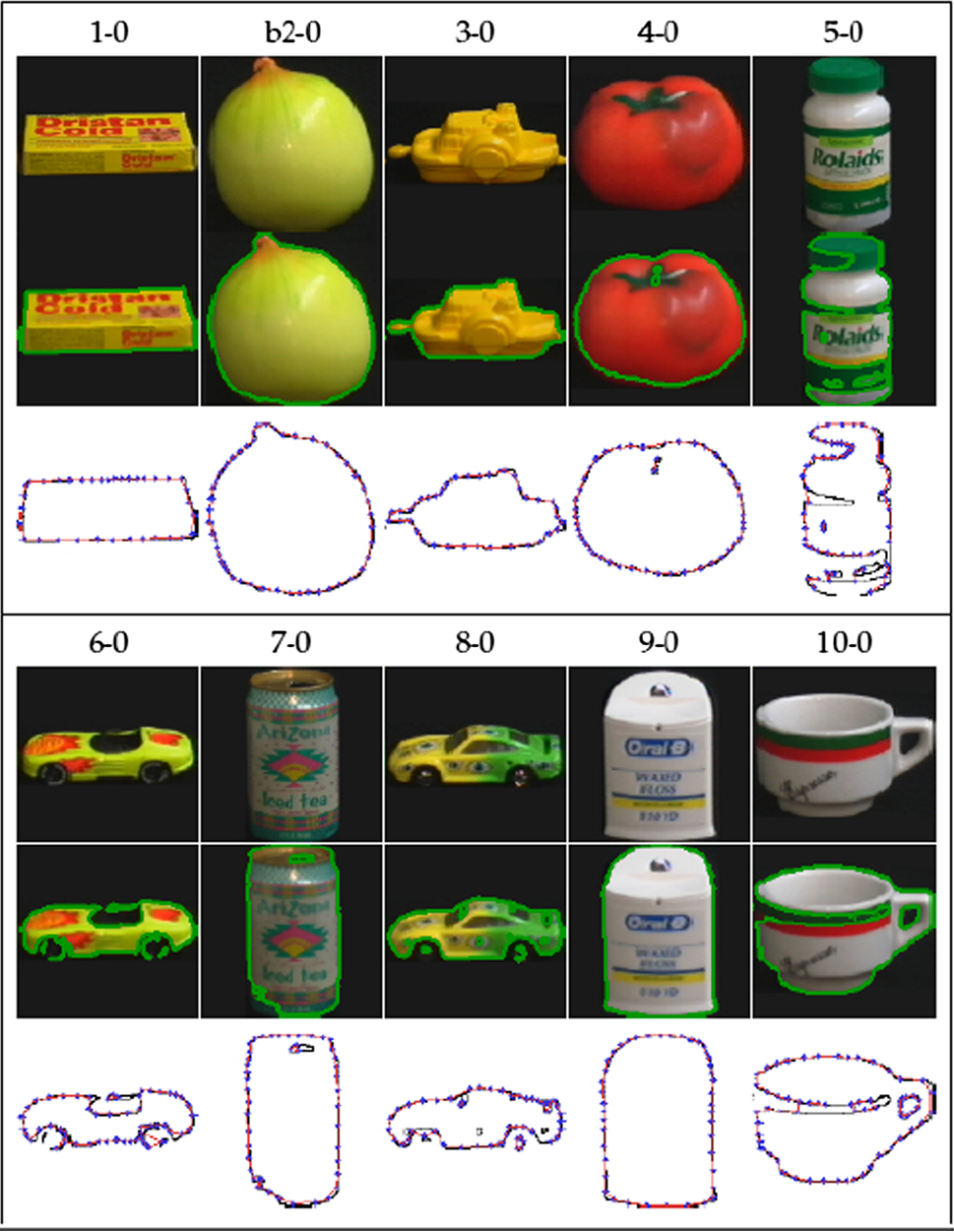
First shape of each of the first 10 objects in coil-100, showing the original image, the thresholded region, and the modified GNG contour representation

**Fig. 3:**
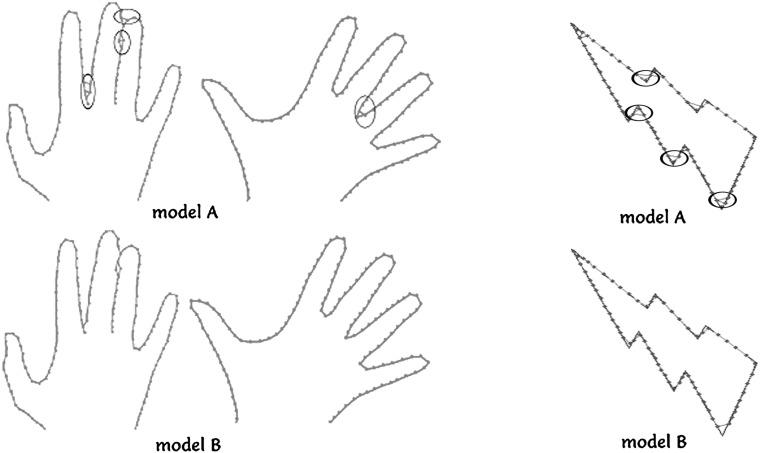
Modification of the GNG network to eliminate multiple connections and to attempt to reduce the network to a single series of sequentially linked nodes. Model A is the original network with the wrong connections (*circled corners*), while model B is our modified network

Figure 2 shows another example of the modified GNG applied to shapes extracted from the Columbia Object Image Library (coil-100) dataset. The 100 object coil-100 dataset consists of colour images of 72 different poses for each object. The poses correspond to $5^{\circ }$ rotation intervals. Figure 3 shows the modified GNG from our own dataset of hands and shapes. Any wrong connections to corners have been accurate eliminated.

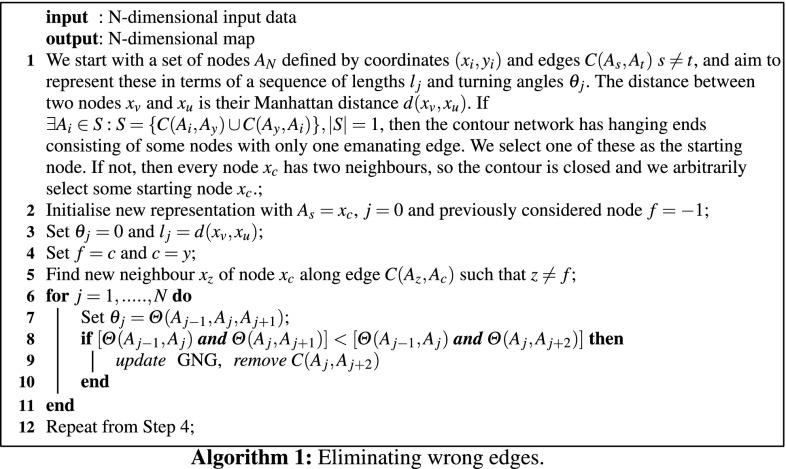



To normalise the graph that represents the contour we must define a starting point, for example the node on the left-bottom corner. Taking that node as the first we must follow the neighbours until all the nodes have been added to the new list. If necessary we must apply a scale and a rotation to the list with respect to the centre of gravity of the list of nodes. We achieved the required alignment by applying a transformation *T* composed by a translation $(t_x,t_y)$, rotation $\theta$, and a scaling *s*. The normalisation is given by Algorithm 2.

6 $$\begin{aligned} T \left[ \begin{array}{r} x_{i}\\ y_{i} \end{array}\right] = \left[ \begin{array}{rr} s(\cos \vartheta )x_{i} & -s(\sin \theta )y_{i}\\ s(\sin \vartheta )x_{i} & s(\cos \theta )y_{i} \end{array}\right] + \left[ \begin{array}{r} t_{x}\\ t_{y} \end{array}\right] \end{aligned}$$

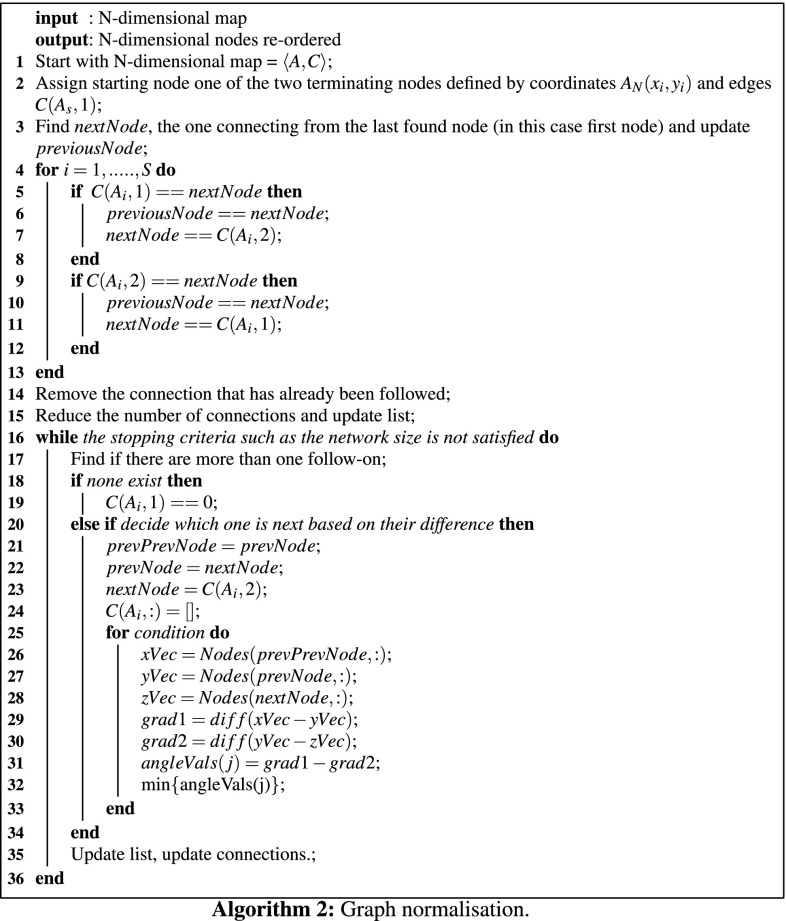


## Adaptive learning

The determination of accurate topology preservation requires the determination of best similarity threshold and best network map without overfitting. Let $\Omega (x)$ denote the set of pixels in the objects of interest based on the configuration of *x* (e.g. colour, texture, etc.) and $\Upsilon$ the set of all image pixels. The likelihood of the required number of nodes to describe the topology of an image *y* is:

7 $$\begin{aligned} p(y|x) & = \left\{ \prod _{u\in \Omega (x)}p_{skin}(u) \prod _{v\in \Upsilon \backslash \Omega (x)}p_{bkgd}(v) \right. \nonumber \\&\quad \left. \propto \prod _{u\in \Omega (x)}\frac{p_{skin}(u)}{p_{bkgd}(u) + p_{skin}(u)} \right\} * e_{T} \end{aligned}$$

and $$e_{T} \le {\prod _{u\in \Omega (x)}p_{skin}(u) + \prod _{v\in \Upsilon \backslash \Omega (x)}p_{bkgd}(v)}$$.

**Fig. 4:**
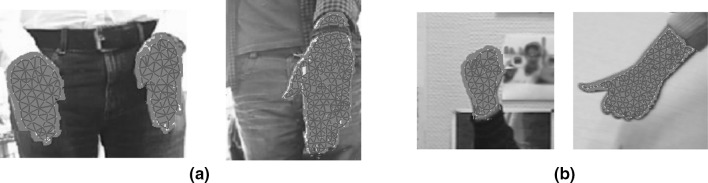
Likelihood node ratios for images with same image resolution but different skin to background ratio. **a** Network adaptation to images of 46,332 pixels with maps of 102 and 162 nodes. **b** Network adaptation to images of 21,903 pixels with maps of 46 and 132 nodes

Figure 4 shows the network map for images with different skin to background ratio.

$e_{T}$ is a similarity threshold and defines the accuracy of the map. If $e_{T}$ is low, the topology preservation is lost and more nodes need to be added. On the contrary, if $e_{T}$ is too big, then nodes have to be removed so that Voronoï cells become wider. For example, let us consider an extreme case where the total size of the image is $I = 100$ pixels and only one pixel represents the object of interest. Let us suppose that we use $e_{T} = 100$ then the object can be represented by one node. In the case where $e_{T} \ge I$ then overfit occurs since twice as many nodes are provided.

In our experiments, the numerical value of $e_{T}$ ranges from $100\le e_{T} \le 900$ and the accuracy depends on the size of the objects’ distribution. The difference between choosing manually the maximum number of nodes and selecting $e_{T}$ as the similarity threshold, is the preservation of the object independently of scaling operations. Algorithm 3 shows the steps of the automatic criterion added to the modified GNG algorithm to minimise user intervention in the learning process.

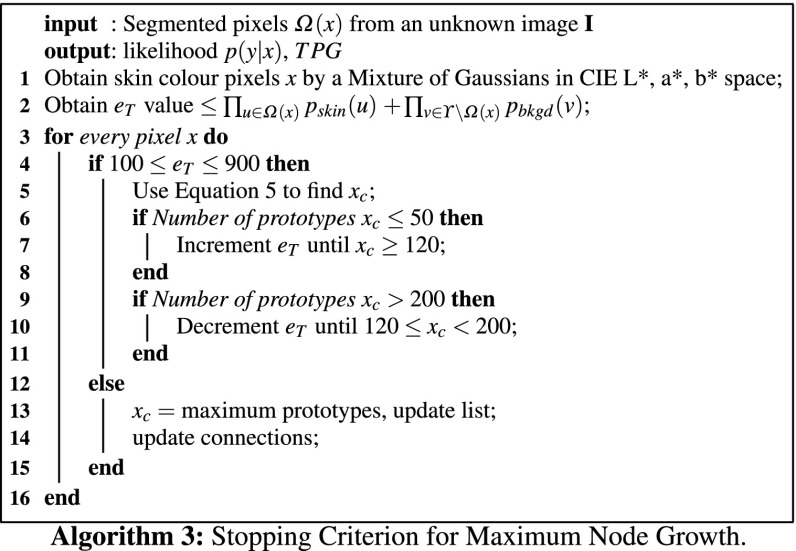



We can describe the optimum number of similarity thresholds, required for the accuracy of the map for different objects, as the unknown clusters *K*, and the network parameters as the mixture coefficients $W_{K}$, with *d*-dimensional means and covariances $\varTheta _{K}$. To do that, we use a heuristic criterion from statistics known as the Minimum Description Length (MDL) [28], which does not require an estimation of the probability *p*(*Y*) as is the case for the *conditional entropy* heuristic criterion [3]. The MDL criterion takes the general form of a prediction error, which consists of the difference between two terms:

8 $$\begin{aligned} E = model\_likelihood - complexity\_term \end{aligned}$$

a likelihood term that measures the model fit and increases with the number of clusters, and a complexity term, used as a penalty, that grows with the number of free parameters in the model. Thus, if the number of cluster is small, we get a low value for the criterion because the model fit is low, while if the number of cluster is large, we get a low value because the complexity term is large.

The information-criterion MDL of Rissanen [28], is defined as:

9 $$\begin{aligned} \hbox {MDL}(K) = -\ln [L(X|W_{K},\varTheta _{K})] + \frac{1}{2}M\ln (N) \end{aligned}$$

where

10 $$\begin{aligned} L(X|W_{K},\varTheta _{K}) = \max \prod _{i=1}^{N}p(x_{i}|W_{K},\varTheta _{K}) \end{aligned}$$

The first term $-\ln [L(X|W_{K},\varTheta _{K})]$ measures the model probability with respect to the model parameter $W_{K},\varTheta _{K}$ defined for a Gaussian mixture by the mixture coefficients $W_{K}$ and *d*-dimensional means and covariances $\varTheta _{K}$. The second term $\frac{1}{2}M\ln (N)$ measures the number of free parameters needed to encode the model and serves as a penalty for models that are too complex. *M* describes the number of free parameters and is given for a Gaussian mixture by $M = 2dK + (K - 1)$ for $(K-1)$ adjustable mixture weights and 2D parameters for *d*-dimensional means and diagonal covariance matrices.

The optimal number of similarity thresholds can be determined by applying the following iterative procedure:

- For all *K*, $$(K_{\min}< K <K_{\max })$$
  (a) Maximize the likelihood $L(X|W_{K},\varTheta _{K})$ using the EM algorithm to cluster the nodes based on the similarity thresholds applied to the dataset.
  (b) Calculate the value of MDL(K) according to Eqs. 9 and 10
- Select the model parameters $(W_{K},\varTheta _{K})$ that correspond to minimisation of the MDL(K) value.

**Fig. 5:**
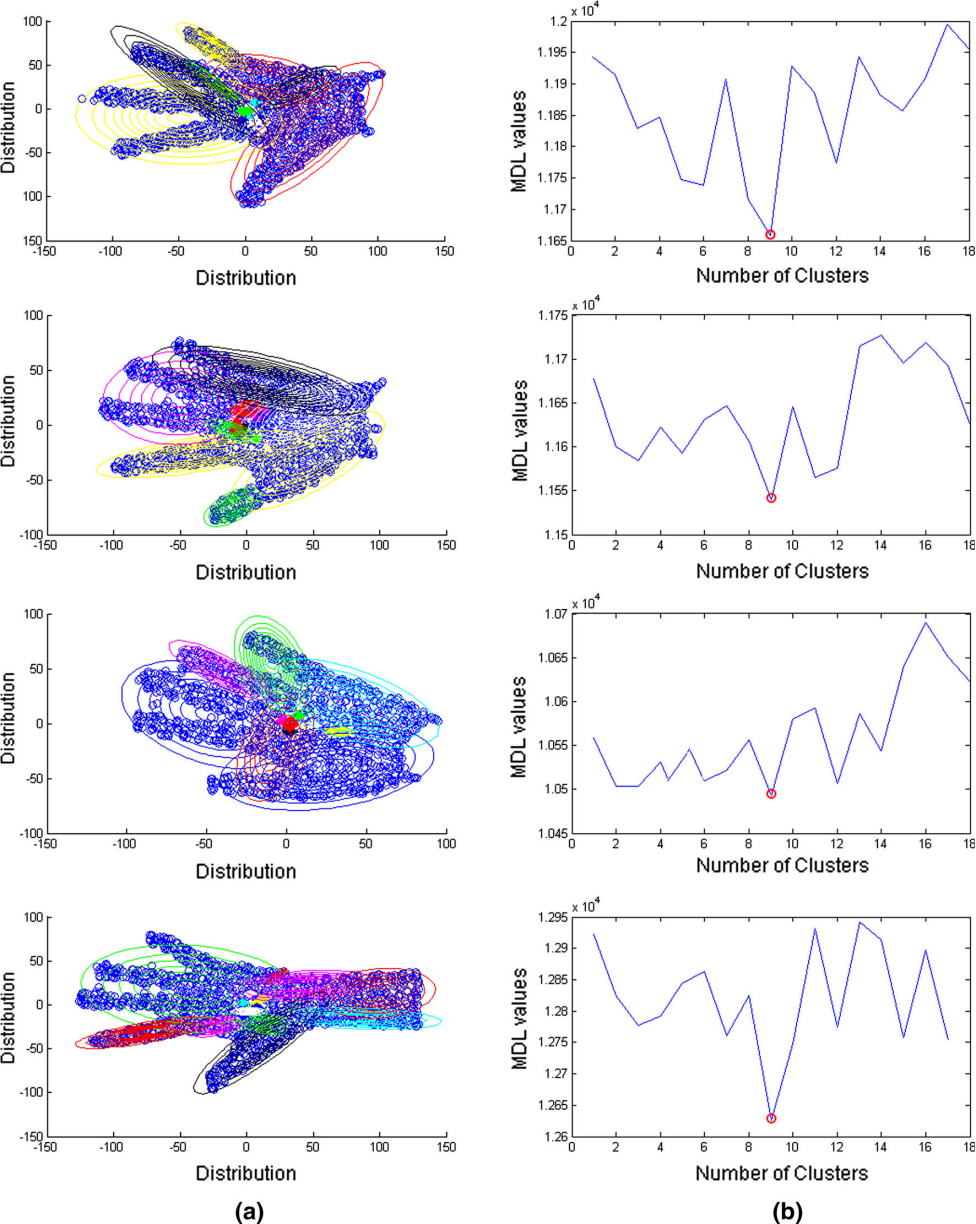
**a** Plot of hand distributions. **b** Plot of the MDL values versus the number of cluster centres. The Minimum Description Length MDL(K) is calculated for all cluster configurations with $(1< K < 18)$ clusters, and a global minimum is determined at 9 (*circled point*)

Figure 5 shows the value of MDL(K) for clusters within the range of $(1< K < 18)$. We have doubled the range in the MDL(K) minimum and maximum values so we can represent the extreme cases of 1 cluster which represents the whole dataset, and 18 clusters which over classify the distribution and corresponds to the overfitting of the network with similarity threshold $e_{T} = 900$. A global minimum and therefore optimal number of clusters can be determined for $K = 9$ which indicates that the best similarity threshold that defines the accuracy of the map without overfitting or underfitting the dataset is $e_{T} = 500$. To account for susceptibility for the *EM* cluster centres as part of the MDL(K) initialisation of the mixture coefficients, the measure is averaged over 10 runs and the minimal value for each configuration is selected. Algorithm 4 summarises the steps.

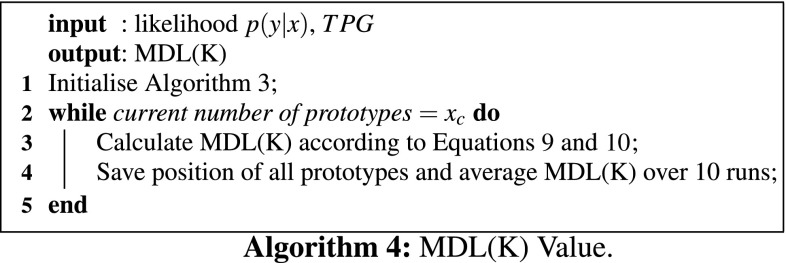



We can now use this optimal network to track objects locally wherever common regions are found. To do that, shape information and colour information from the 1*st* and any subsequent frames are added to the *TPG* map and can be used for the learning in a sequence of *k* frames. The segmented frame and the stored shape and colour information in each node is given by:

11 $$\begin{aligned} S(x;P(g(x,y);t) = p(k|x) \propto P(g(x,y),t-1), TPG_{t-1} \end{aligned}$$

**Fig. 6:**
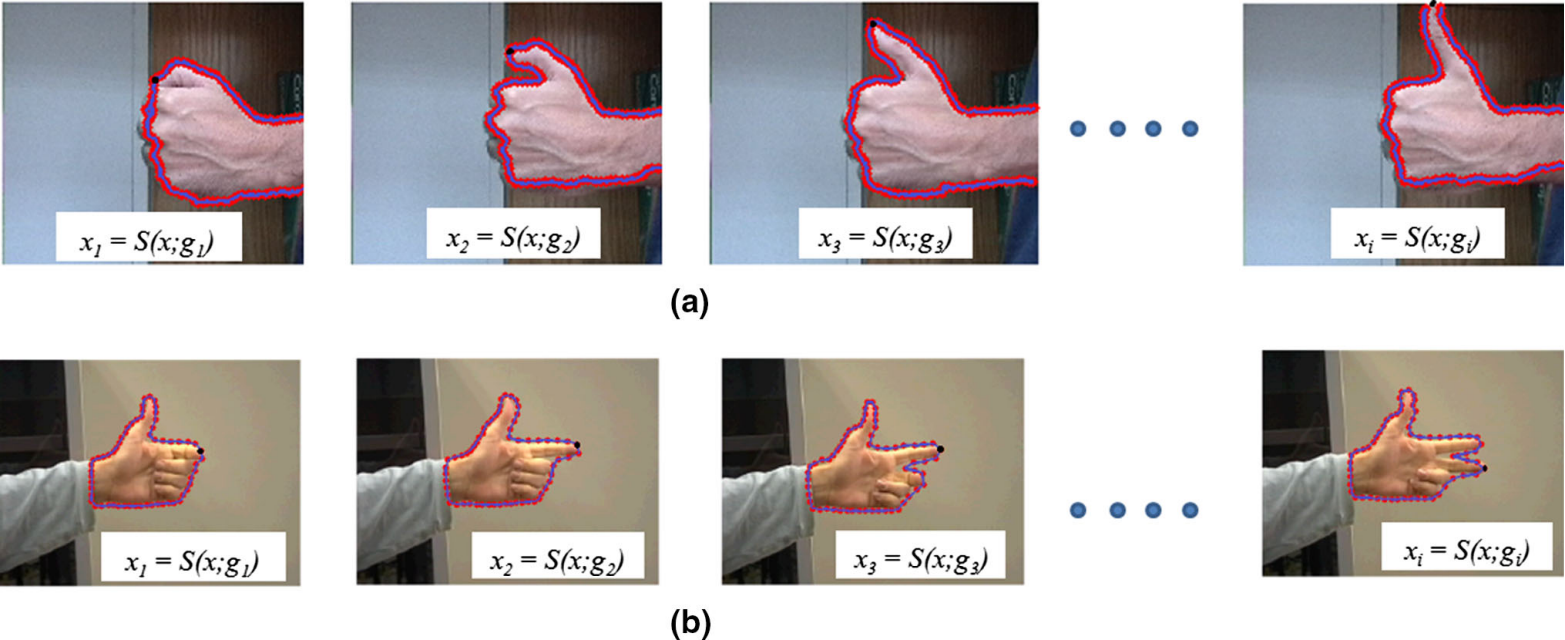
Network convergence for two sets of images after a sequence of *k* frames. The network is defined by the shape ${S}(x;P(g(x,y)))$ and the movement of the nodes depend on the posterior probability *P*(*g*(*x*, *y*)). The higher the probability of a node to belong to the skin prior probability, the faster the node will re-adjust its position to the new input distribution (*black dot*)

Figure 6 shows the convergence of the network with shape and posterior probability per node.

## Experiments

In this section, different experiments are shown validating the capabilities of our extended GNG method to represent 2D and 3D hand models. The proposed method by considering elimination of noisy connections during the learning process is able to define an optimal number of nodes using the MDL criterion. The method has also been used in 3D datasets. First, a quantitative study is performed adding different levels of noise to the ground truth model (datasets). Using the ground truth models and the generated ones adding noise, we are able to measure the error produced by our method. In addition, our method is compared against the state-of-the-art algorithms Active Shape Models and Poisson surface reconstruction.

All methods have been developed and tested on a desktop machine of 2.26 GHz Pentium IV processor. These methods have been implemented in MATLAB and C++. The Poisson surface reconstruction method has been implemented using the PCL library^3^ [29].

### Benchmark data

**Fig. 7:**
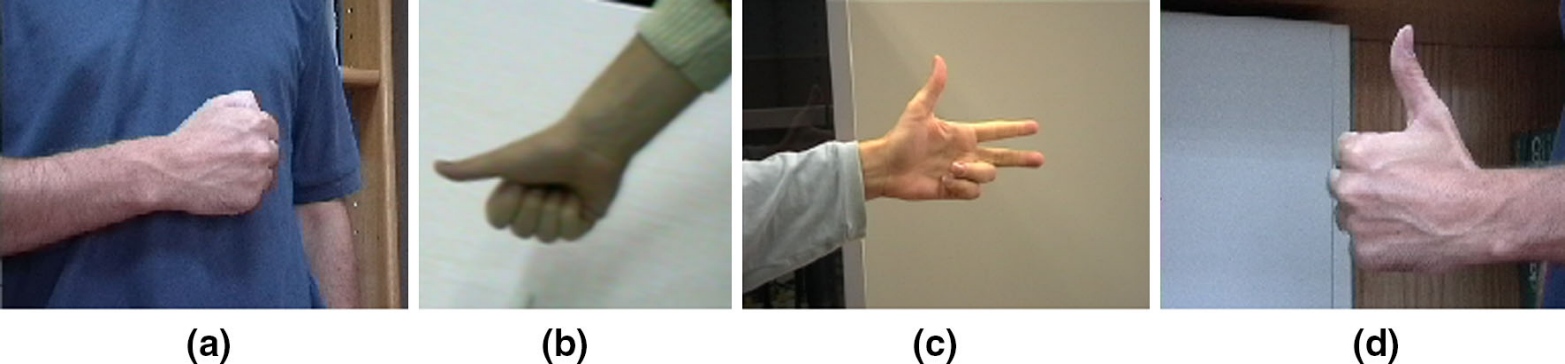
Some common gestures used in sign language

We tested our modified GNG network on a dataset of hand images recorded from 5 participants each performing different gestures (Fig. 7) that frequently appear in sign language. To create this dataset, we have recorded images over several days and a simple webcam was used with image resolution $800 \times 600$. In total, we have recorded over 12000 frames, and for computational efficiency, we have resized the images from each set to $300 \times 225$, $200 \times 160$, $198 \times 234$, and $124 \times 123$ pixels. We obtained the dataset from the University of Alicante, Spain and the University of Westminster, UK. Also, we tested our method with 49 images from Mikkel B. Stegmann^4^ online dataset. In total we have run the experiments on a dataset of 174 images. Since the background is unambiguous, the network adapts without occlusion reasoning. For our experiments, only complete gesture sequences are included. There are no gestures with partial or complete occluded regions, which means that we do not model multiple objects that interact with the background.

Furthermore, we have performed the experiments having in mind specific applications, thus limiting its applicability. The quality and stability of the results at close range makes it worthwhile for webcam or green screen sign language applications which share a close range viewing distance and a relatively uncluttered background.

**Fig. 8:**
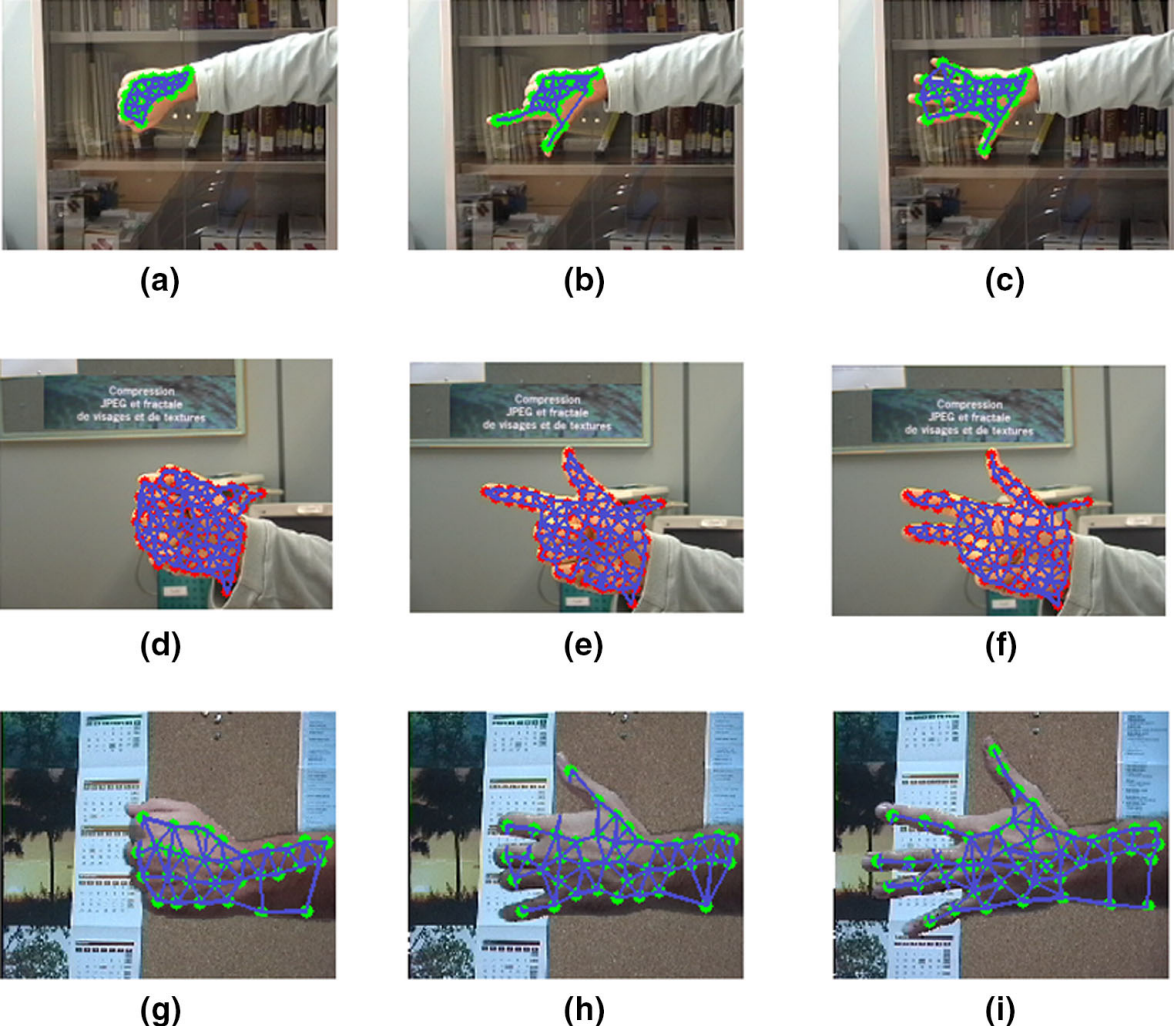
Examples of gestures in three different cluttered backgrounds

We have also tested the system in a more generic background where shadows, changes in lighting and extremely cluttered backgrounds are common. Figure 8 shows that when colour information is incorporated into the network, the system is able to represent the gesture and only a few nodes adjust to nearby similar pixels. Gesture representation is possible as long as no homogeneity is applied around the gesture.

**Fig. 9:**
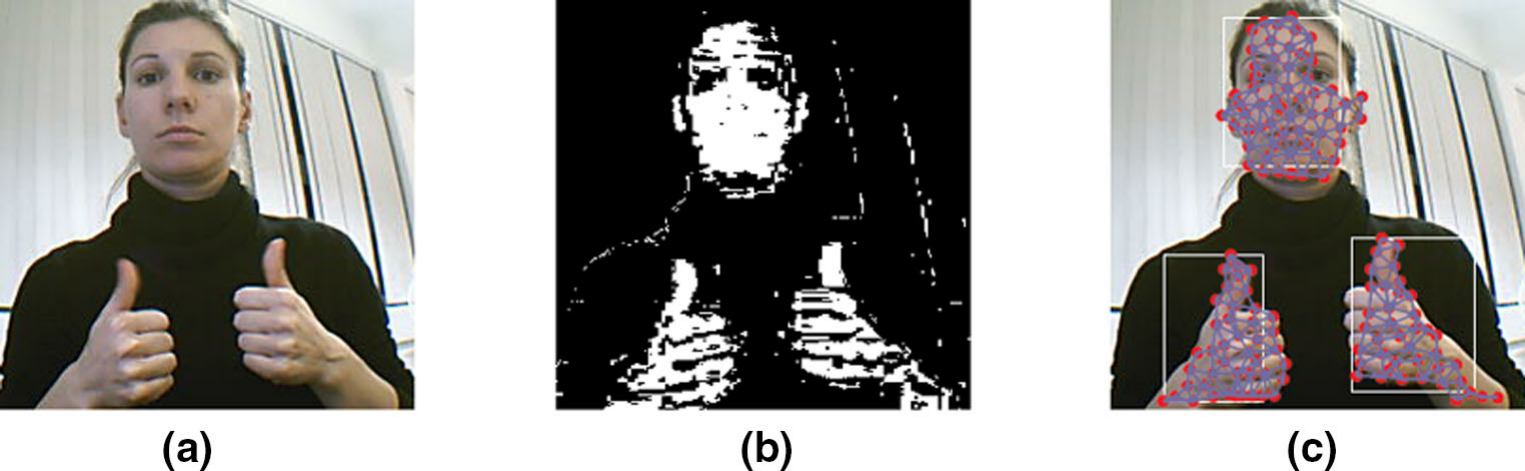
Example of correctly detected hands and face based on the golden ratio regardless of the scale and the position of the hands and the face. **a** Original image, **b** after applying EM to segment skin region, and **c** hand and face detector taking into account the connected nodes in the networks as well as the percentage of skin in the rectangular area

To classify a region as a hand or face, we take into account domain knowledge information that always respects some proportions found in hands and human faces [11]. To do that we find the centroid, height and width of the connected nodes in the networks as well as the percentage of skin in the rectangular area (Fig. 9). Since the height to width ratio for hands and human faces fall into a small range, we are able to reject or accept if the topology of a network does or does not represent a hand. Studies [7, 11] have shown that the height to width ratio of human face and hands fall within a range based on the well known Golden Ratio (Eq. 12). Thus, we consider a network as a hand or not if the height to width ratio of the region falls within a range of the Golden Ratio ± Tolerance. In the case where the hand is in a folded posture the rule still applies but with different percentage for the *Tolerance*. The values for the *Tolerance* were found by experimentation, and range from 0.5 to 0.7 based on the hand posture.

12 $$\begin{aligned} \varphi \equiv \frac{\hbox {Height}}{\hbox {Width}} \equiv \frac{(1 + \sqrt{5})}{2} \end{aligned}$$

Table 2 shows topology preservation, execution time, and number of nodes when different variants in the $\lambda$ and the *K* are applied in the gesture (d) from Fig. 7 as the input space. Faster variants get worse topology preservation but the network converges quickly. However, the representation is sufficient and can be used in situations where minimum time is required like online learning for detecting obstacles in robotics where you can obtain a rough representation of the object of interest in a given time and with minimum quality.

**Table 2:** Topology preservation and processing time using the quantisation error and the topology preservation error for different variants

Variant	Number of nodes	Time (s)	QE	TE
$\text {GNG}_{\lambda = 100, K=1}$	23	0.22	8.932453	0.4349
$\hbox {GNG}_{\lambda = 100, K=9}$	122	0.50	5.393949	−0.3502
$\hbox {GNG}_{\lambda = 100, K=18}$	168	0.84	5.916987	−0.0303
$\hbox {GNG}_{\lambda = 300, K=1}$	23	0.90	8.024549	0.5402
$\hbox {GNG}_{\lambda = 300, K=9}$	122	2.16	5.398938	0.1493
$\hbox {GNG}_{\lambda = 300, K=18}$	168	4.25	4.610572	0.1940
$\hbox {GNG}_{\lambda = 600, K=1}$	23	1.13	0.182912	−0.0022
$\hbox {GNG}_{\lambda = 600, K=9}$	122	2.22	0.172442	0.3031
$\hbox {GNG}_{\lambda = 600, K=18}$	168	8.30	0.169140	−0.0007
$\hbox {GNG}_{\lambda = 1000, K=1}$	23	1.00	0.188439	0.0750
$\hbox {GNG}_{\lambda = 1000, K=9}$	122	12.02	0.155153	0.0319
$\hbox {GNG}_{\lambda = 1000, K=18}$	168	40.98	0.161717	0.0111

**Fig. 10:**
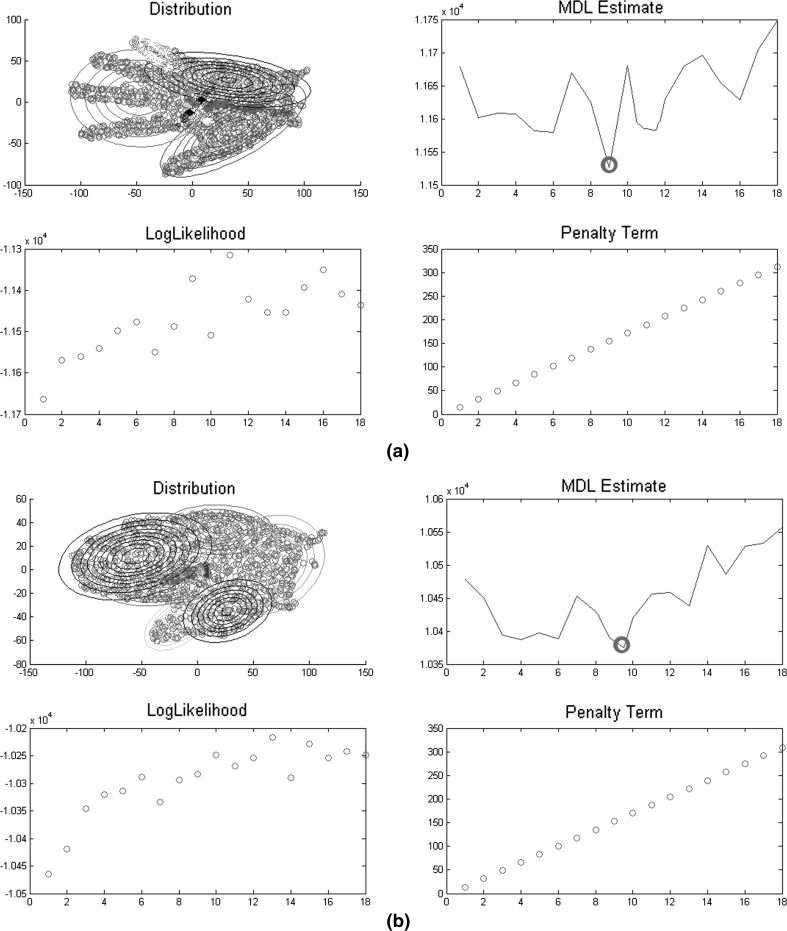
**a**, **b** Distribution of two different hand shapes with plotted MDL(K) values within the range of $(1< K < 18)$ and a global minimum at 9 (*circled point*). **a**, **b** also show the likelihood term that measures the model fit and the penalty; both of which grow with the number of used clusters

Figure 10 shows the distribution of two different hand shapes and the plots of the MDL(K) cluster centres within the range of $(1< K < 18)$. The optimum cluster is achieved at $K = 9$ (circled point).

**Fig. 11:**
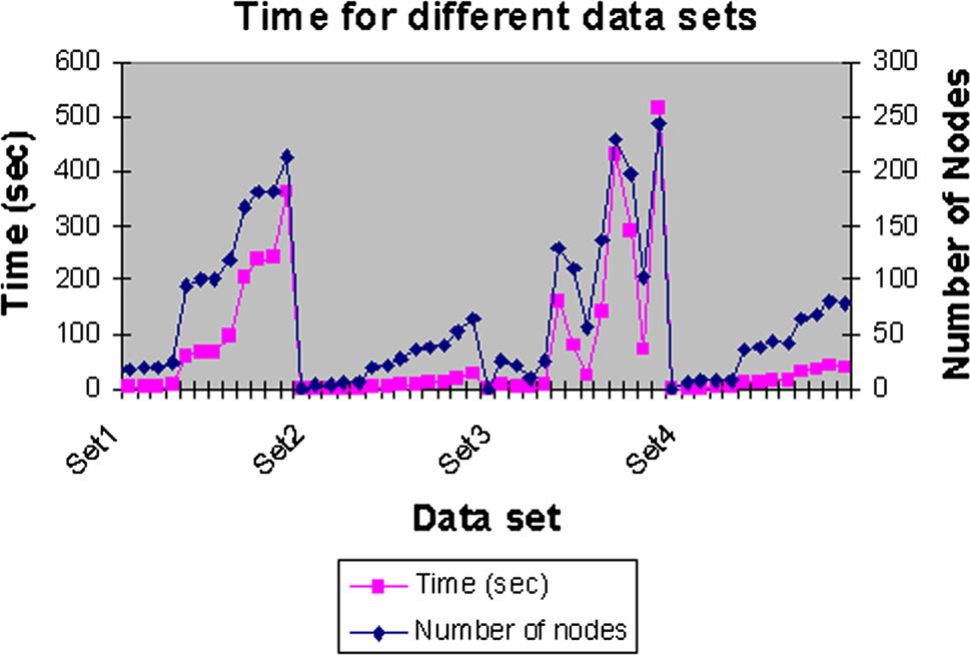
Time taken to insert the maximum number of nodes per dataset

Table 3 shows the topology preservation error for a number of nodes. We can see that the insertion of more nodes makes no difference to the object’s topology. Based on the maximum size of the network, an optimum result is achieved when at least half of the network is developed. Table 3 shows that for the different type of gestures, this optimum number is in the range >90 and <130. Furthermore, the more nodes added during the learning process, the more time it takes for the network to grow (Fig. 11).

**Table 3:** The topology preservation error for gestures (a–d)

Image (a)		Image (b)		Image (c)		Image (d)	
Nodes	TE	Nodes	TE	Nodes	TE	Nodes	TE
26	−0.0301623	26	−0.021127	24	−0.017626	19	−0.006573
51	−0.030553	51	−0.021127	47	−0.047098	37	−0.007731
77	0.04862	77	0.044698	71	0.046636	56	0.027792
102	0.048256	102	0.021688	95	0.017768	75	0.017573
128	0.031592	128	0.011657	119	0.014589	94	0.018789
153	0.038033	153	0.021783	142	0.018929	112	0.016604
179	0.047636	179	0.017223	166	0.017465	131	0.017755
205	0.038104	205	−0.013525	190	0.017718	150	0.007332
230	0.037321	230	0.017496	214	−0.007543	168	0.007575

**Fig. 12:**
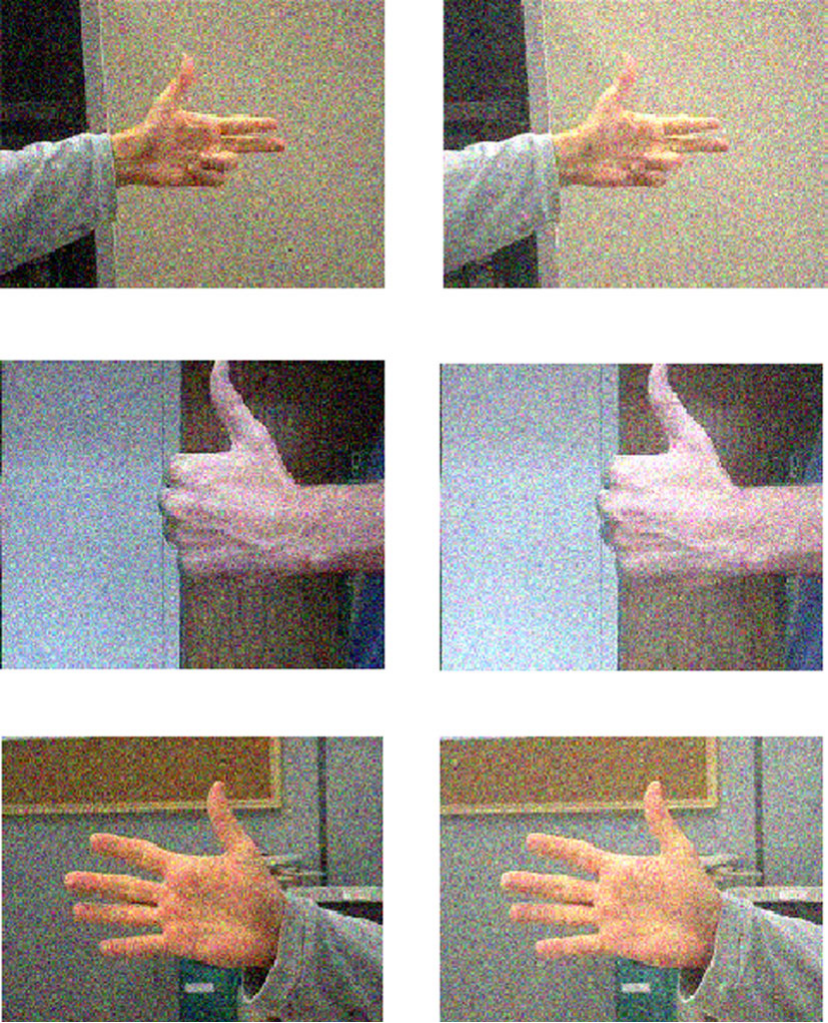
Gestures with different levels of Gaussian noise. From *left* to *right* mean = 0, sigma = 0; mean = 0, sigma = 0.25

Finally, we added different levels of Gaussian noise to three different gestures to test the validity of the modified GNG in comparison with Kohonen map and the growing cell structures (GCS). The results of applying different levels of noise to the gestures are shown in Fig. 12, and error measurements for all methods are calculated in Table 4.

**Table 4:** Error measurements for modified GNG, Kohonen and GCS

Gestures	Method	Nodes	RMS	TE
Gesture-three fingers (sigma = 0)	Modified GNG	21	**0.2558**	**0.055554**
Gesture-three fingers (sigma = 0)	Kohonen	25	1.6410	0.172629
Gesture-three fingers (sigma = 0)	GCS	30	0.5494	0.159913
Gesture-three fingers (sigma = 0.25)	Modified GNG	21	**1.4189**	**0.083485**
Gesture-three fingers (sigma = 0.25)	Kohonen	25	2.6578	0.237586
Gesture-three fingers (sigma = 0.25)	GCS	30	1.6134	0.241429
Gesture-thumb (sigma = 0)	Modified GNG	25	**0.2440**	**0.046621**
Gesture-thumb (sigma = 0)	Kohonen	30	0.5376	0.194685
Gesture-thumb (sigma = 0)	GCS	31	0.3144	0.176336
Gesture-thumb (sigma = 0.25)	Modified GNG	25	**0.3844**	**0.058153**
Gesture-thumb (sigma = 0.25)	Kohonen	30	0.6956	0.242131
Gesture-thumb (sigma = 0.25)	GCS	31	0.3956	0.239292
Gesture-open hand (sigma = 0)	Modified GNG	23	** 0.9660**	**0.048011**
Gesture-open hand (sigma = 0)	Kohonen	25	3.4727	0.146884
Gesture-open hand (sigma = 0)	GCS	27	2.3790	0.150354
Gesture-open hand (sigma = 0.25)	Modified GNG	23	** 1.4025**	**0.059658**
Gesture-open hand (sigma = 0.25)	Kohonen	25	3.5340	0.240014
Gesture-open hand (sigma = 0.25)	GCS	27	2.4599	0.112732

Bold numbers demonstrate the lowest errors for our modified GNG network

### Variability and comparison with the snake model

**Fig. 13:**
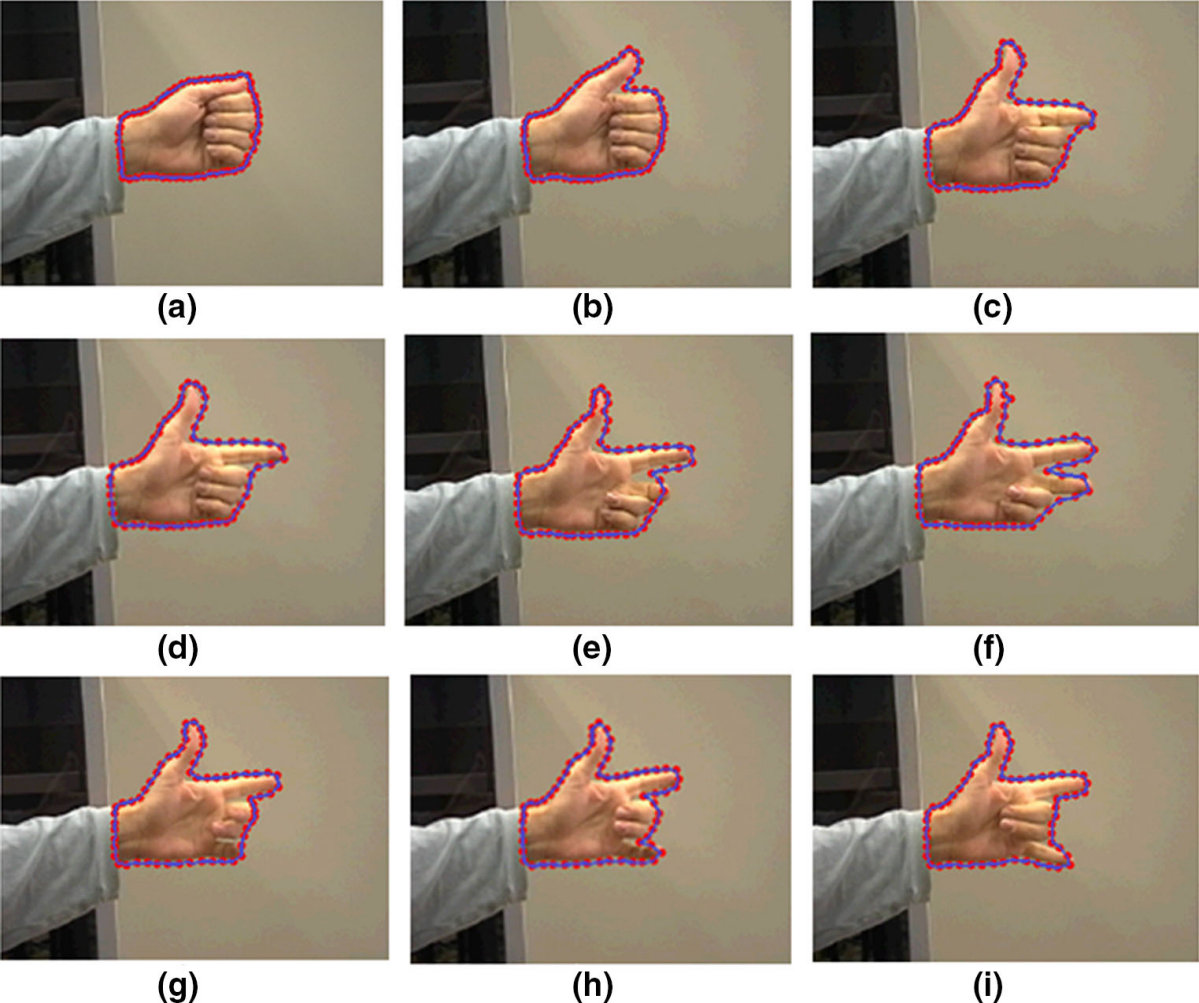
Tracking a gesture. The images correspond from *left* to *right* and from top to bottom to every 10th frame of a 190 frame sequence. In each image the *red points* indicate the nodes and their adaptation after 4 iterations (colour figure online)

Our modified GNG network has been compared to the methodology of the active snake model. The snake converges when all the forces achieve an equilibrium state. The drawbacks with this method are that the snake has no a priori knowledge of the domain, which means it can deform to match any contour; this attribute is not desirable if we want to keep the specificity of the model or preserve the physical attributes such as geometry, topological relations, etc., and that the *active* step is performed globally even if parts of the snake have already converged. Figure 13 shows the tracking of a hand gesture using the modified GNG in the outline of the hand.

**Fig. 14:**
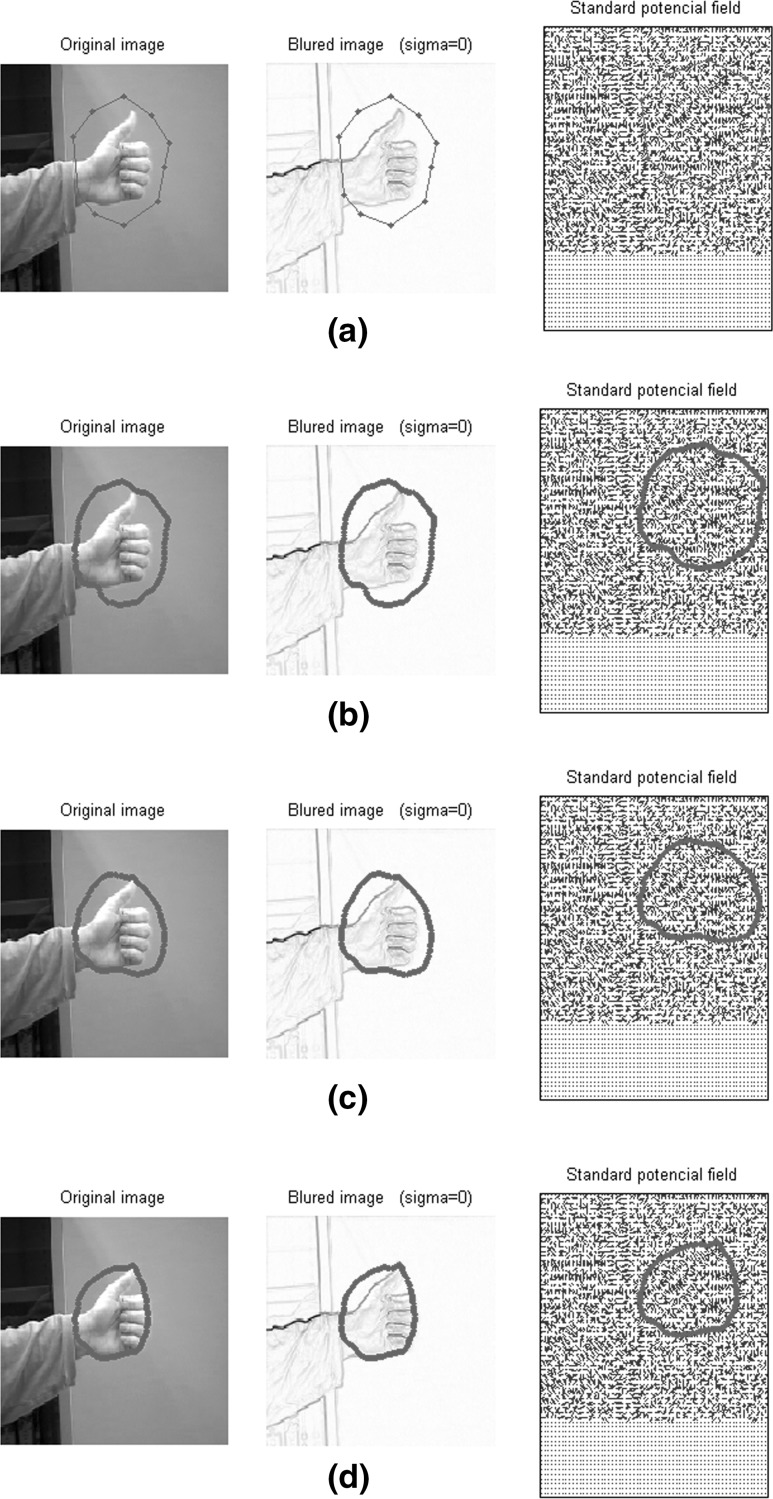
**a** Manual initialisation of the snake. **b**–**d** Adaptation of the snake after a number of iterations

Figure 14 shows the fitting results of a snake applied to the same gesture. Figure 14a is the original state of the snake after manually locating an area around the hand. The closer we allocate landmark points around the hand the faster the convergence of the snake. The snake after a number of iterations converges to the palm of the hand but fails to converge around the thumb.

The parameters for the snake are summarised in Table 5. The execution time for modified GNG is approximately 4 times less compared to the snake. The computational and convergence results are summarised in Table 6.

**Table 5:** Parameters and performance for snake

Hand	Constants	Iterations	Time (s)
Sequence (a)	$\alpha = 0.05$	40	15.29
	$$\beta = 0$$		
	$$\gamma = 1$$		
	$$\kappa = 0.6$$		
	$$D_{\min} = 0.5$$		
	$$D_{\max } = 2$$		
Sequence (b)	$\alpha = 4$	50	15.20
	$$\beta = 1$$		
	$$\gamma = 2$$		
	$$\kappa = 0.6$$		
	$$D_{\min} = 0.5$$		
	$$D_{\max } = 2$$		
Sequence (c)	$\alpha = 4$	40	12.01
	$$\beta = 1$$		
	$$\gamma = 3$$		
	$$\kappa = 0.6$$		
	$$D_{\min} = 0.5$$		
	$$D_{\max } = 2$$		
Sequence (d)	$\alpha = 4$	20	5.60
	$$\beta = 1$$		
	$$\gamma = 3$$		
	$$\kappa = 0.6$$		
	$$D_{\min} = 0.5$$		
	$$D_{\max } = 2$$

**Table 6:** Convergence and execution time results of modified GNG and snake

Method	Convergence (iteration times)	Time (s)
Snake	20	5.60
	40	12.01
	50	15.20
	40	15.29
Modified GNG	2	0.73
	2	1.22
	3	2.17
	5	4.88

### 3D reconstruction

This section shows the result of applying an existing approach proposed by Orts-Escolano et al. [25] for performing 3D surface reconstruction using the GNG algorithm. In this work, we focused on the application of the above-mentioned method for performing reconstruction of human hands and faces that were acquired using the Kinect sensor. Moreover, some experiments were performed using synthetic data.

In [25], the original GNG algorithm is extended to perform 3D surface reconstruction. Furthermore, it considers surface normal information during the learning process. It modifies original Competitive Hebbian Learning process, which only considered the creation of edges between neurons, producing wire-frame 3D representations. Therefore, it is necessary to modify the learning process in order to create triangular faces during network adaptation.

The edge creation, the neurons insertion and the neuron removal stages were extended considering the creation of triangular faces during this process. Algorithm 5 describes the extended CHL to produce triangular faces during the adaption process.

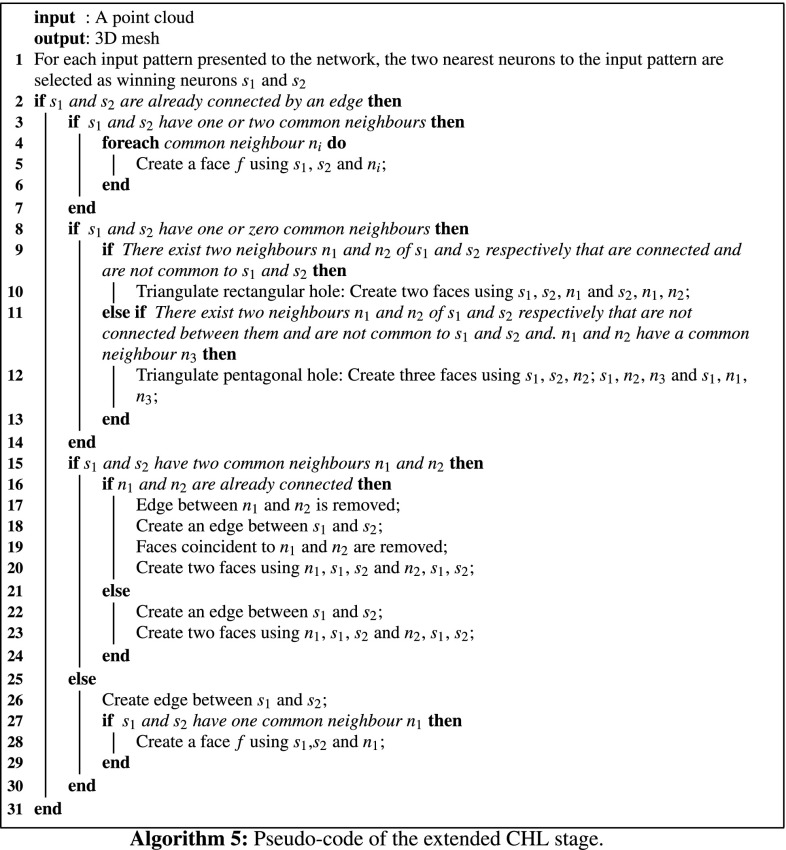



**Fig. 15:**
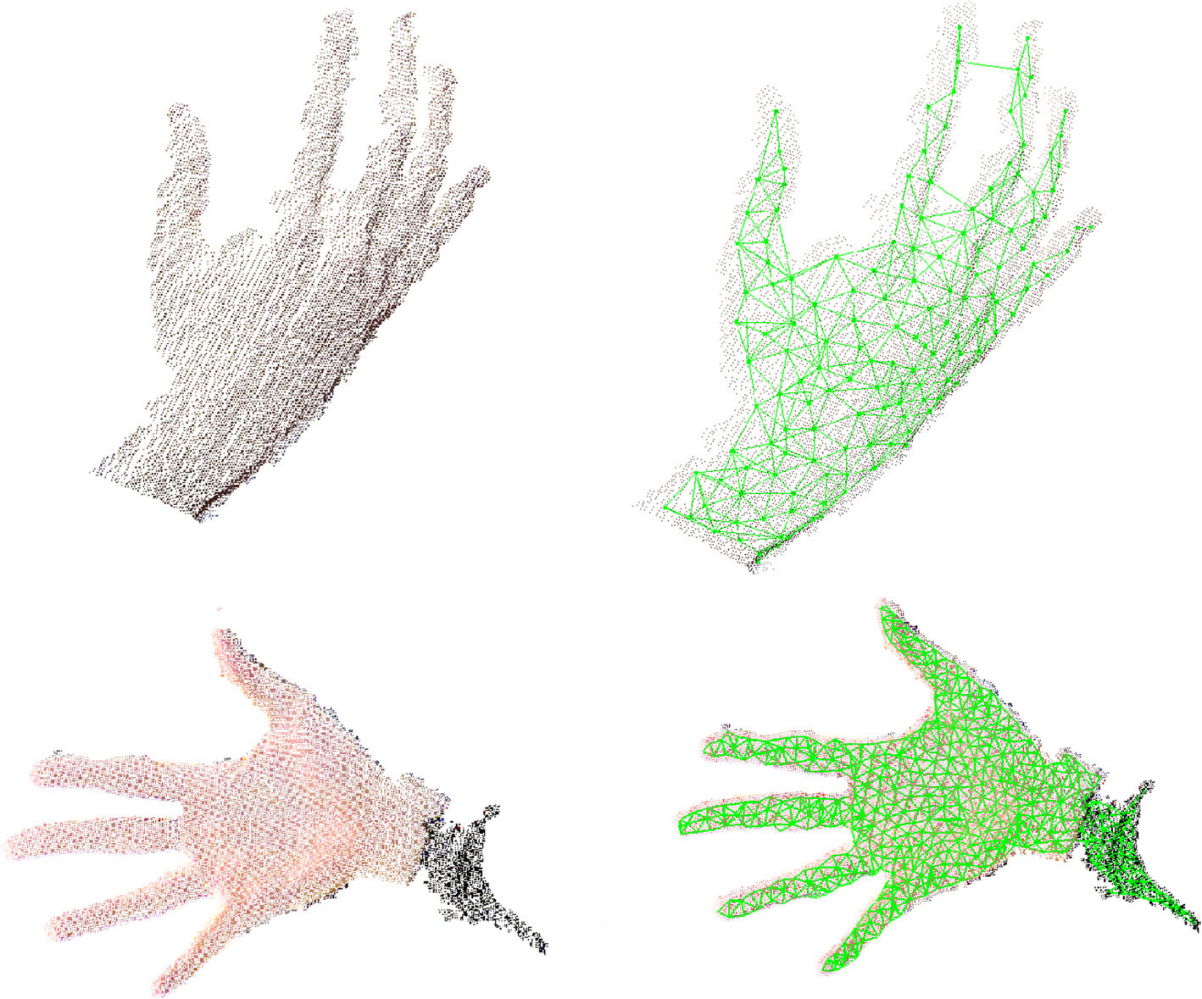
The two images on the left represent the raw data obtained from the low-cost sensor Kinect. The wire-frame representation generated by the original GNG is shown on the right

Figure 15 shows the model created by applying the original GNG algorithm using as an input data a point cloud obtained using the Kinect sensor. It can be appreciated how the GNG produces a wire-frame representation of the input data, but no information about 3D surfaces is provided.

**Fig. 16:**
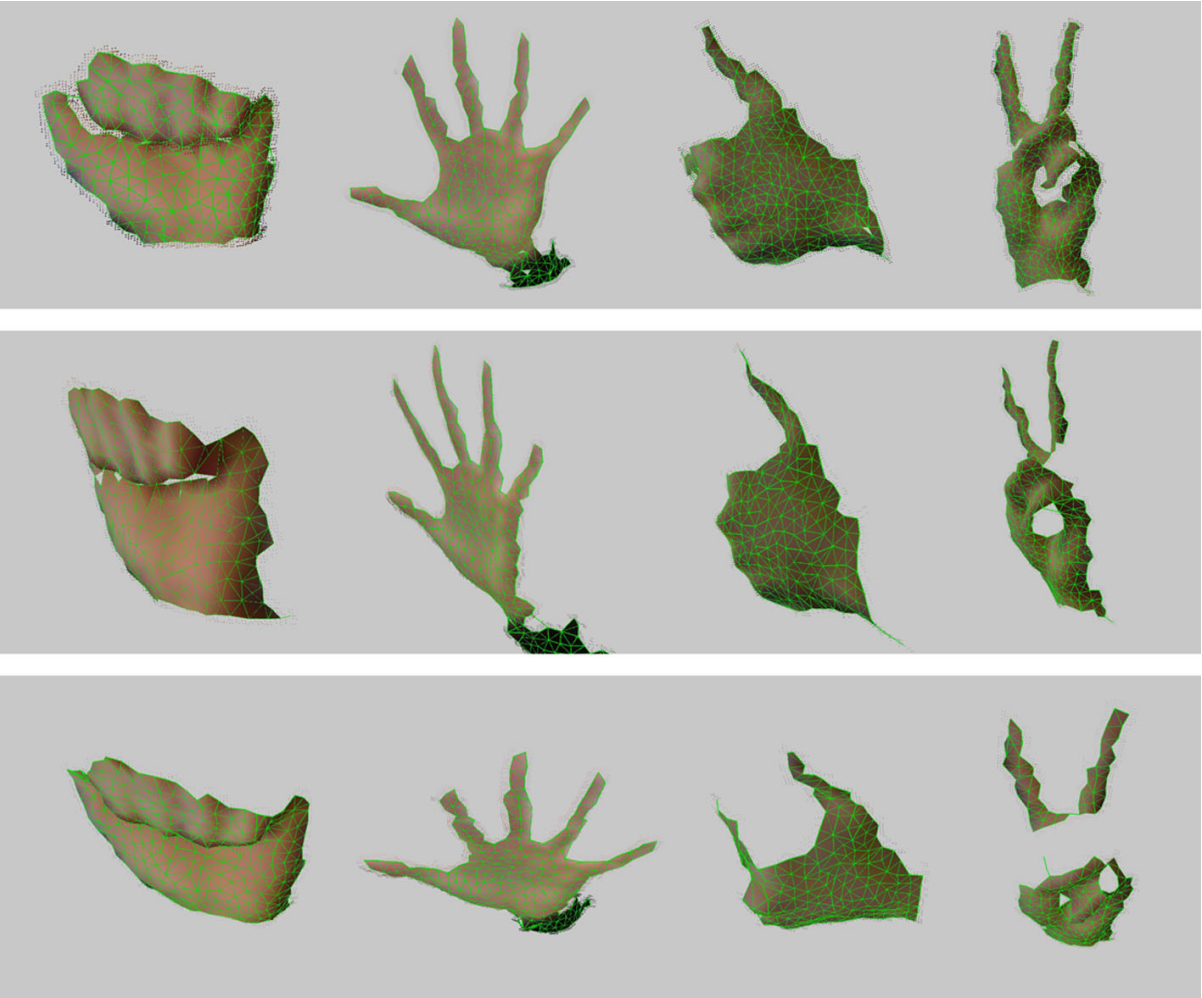
GNG 3D surface reconstructions. 3D reconstruction of different hand poses obtained using the Kinect sensor

Figure 16 shows the 3D mesh created using the method mentioned above. It can be seen how this extended algorithm is able to create a coloured 3D mesh, surface information, that represents the input data. Since point clouds obtained using the Kinect are partial 3D views, the mesh obtained is not complete and therefore the model generated by the GNG is an open coloured mesh.

Moreover, it can also be appreciated that the generated representation is accurate and implicitly it performs some typical computer vision preprocessing steps such as filtering, downsampling and 3D reconstruction.

**Fig. 17:**
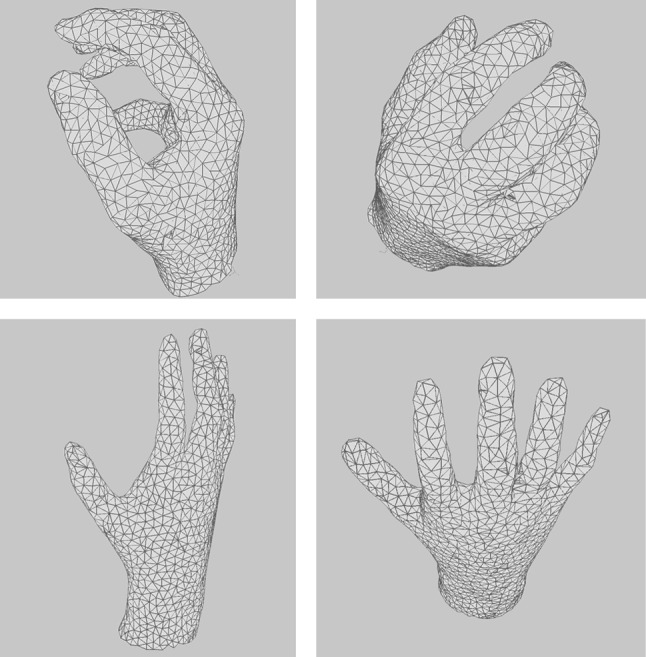
GNG 3D surface reconstructions. 3D reconstruction of two 3D hand models generated using CAD software

Figure 17 shows the result of applying the GNG-based method for surface reconstruction applied to complete hand 3D models. These models were synthetically generated using 3D CAD software.

**Fig. 18:**
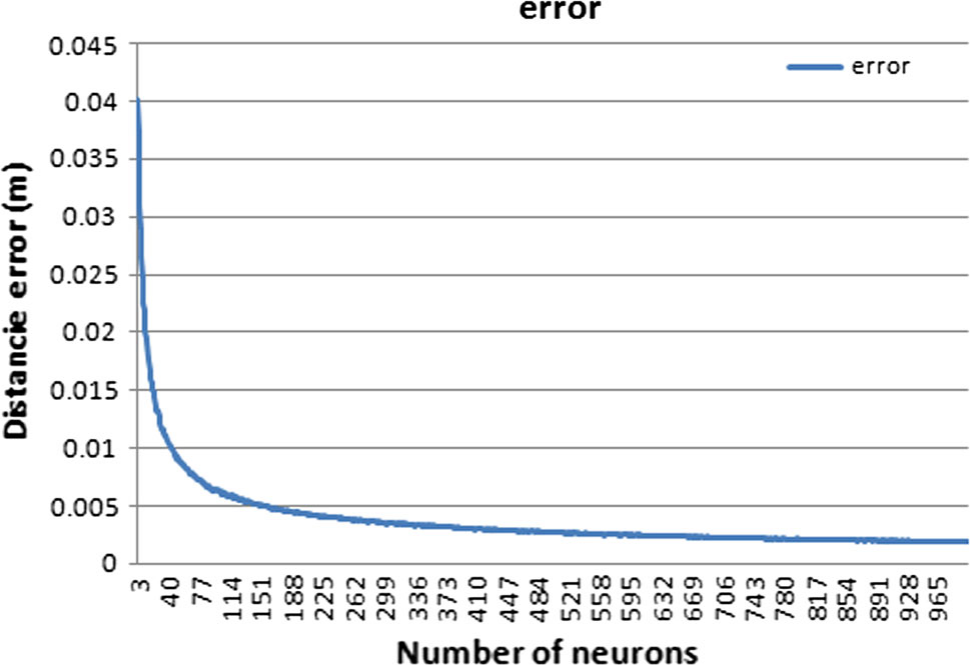
Mean error based on the number of neurons that compose the network

Figure 18 shows the mean square error of different representations of the hand obtained with different numbers of neurons. In addition, the graph shows that with approximately 180 neurons, the adaption error obtained is satisfactory and provides an adequate representation of the input data. We chose the minimum number of neurons with an acceptable quality as it allows real-time processing.

**Fig. 19:**
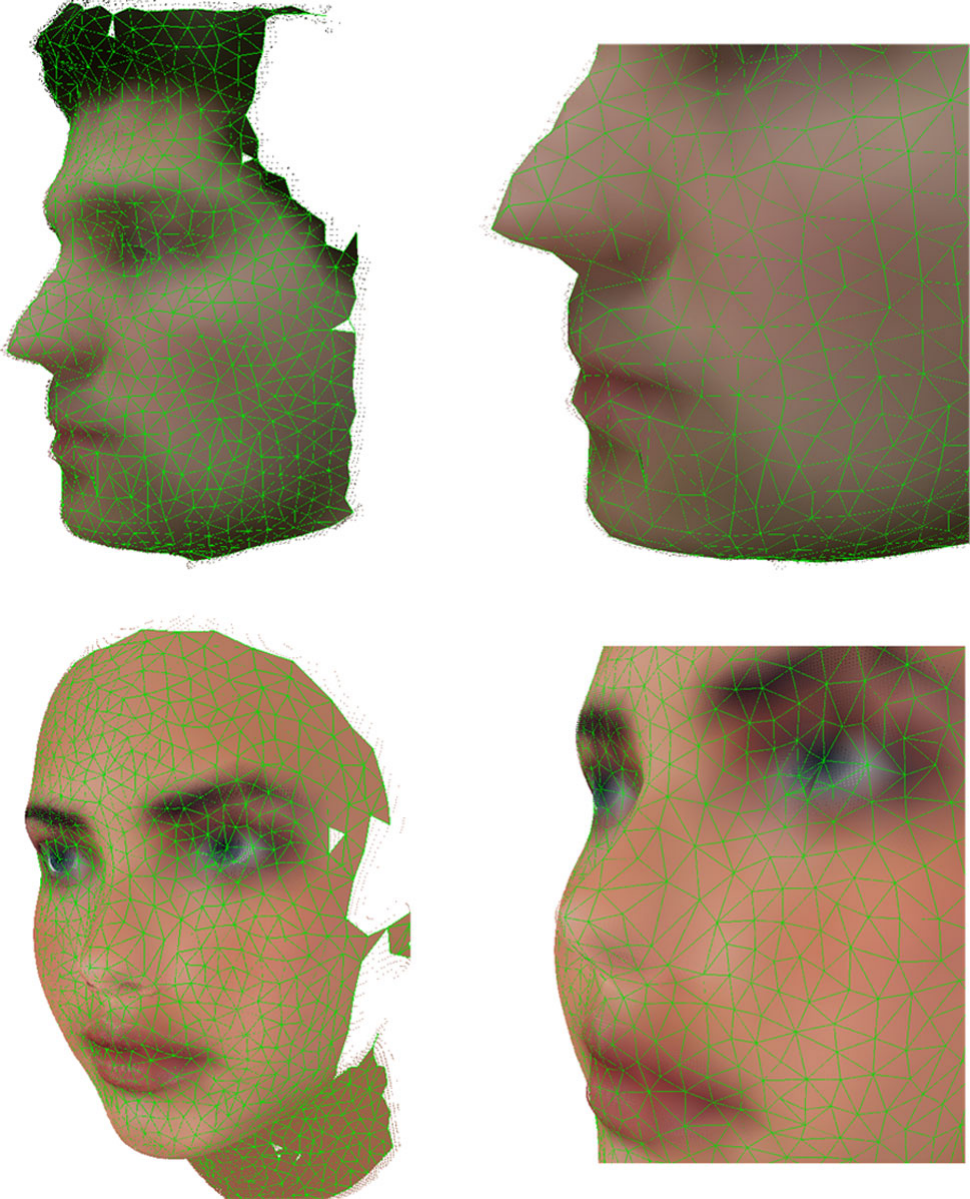
GNG 3D reconstructions. *Top* 3D face reconstruction from data obtained using the Kinect sensor. *Bottom* 3D face reconstruction from data synthetically generated using the Blensor software

Finally, we performed some experiments using 3D human faces instead of hands to demonstrate that the method can also deal with different shapes. Figure 19 shows the 3D reconstruction of a human face acquired using the Kinect sensor (top) and the 3D reconstruction of a synthetically generated human face (bottom). Both faces were reconstructed using the GNG for 3D surface reconstruction. Synthetic data were generated using the Blensor software [12], for simulating a virtual Kinect sensor (noise-free).

In all our experiments, the parameters of the network are as follows: $\lambda = 100$ to 1000, $\epsilon _x = 0.1$, $\epsilon _n = 0.005$, $\Delta x_{s_{1}} = 0.5$, $\Delta x_{i} = 0.0005$, $\alpha _{\max } = 125$.

While 3D downsampling and reconstruction methods like Poisson or Voxelgrid are not able to deal with noisy data, GNG method is able to avoid outliers and obtain an accurate representation in presence of noise. This ability is due to the Hebbian learning rule used and its random nature that update vertex location based on the average influence of a large number of input patterns.

## Conclusions and future work

Based on the capabilities of GNG to readjust to new input patterns without restarting the learning process, we developed an approach to minimise the user intervention by utilising an automatic criterion for maximum node growth. This automatic criterion for GNG is based on the object’s distribution and the similarity threshold ($e_{T}$) which determines the preservation of the topology. The model is then used for the representation of motion in image sequences by initialising a suitable segmentation. During testing we found that for different shapes there exists an optimum number that maximises topology learning versus adaptation time and MSE. This optimal number uses knowledge obtained from information-theoretic considerations. Furthermore, we have shown that the low dimensional incremental neural model (GNG) adapts successfully to the high dimensional manifold of the hand by generating 3D models from raw data received from the Kinect. Future work will aim at improving system performance at all stages to achieve a natural user interface that allows us to interact with any object manipulation system. Likewise, the acceleration of the whole system should be completed on GPUs.

## Footnotes

1 Webcam with image resolution $800\times 600$.

2 Kinect for XBox 360: http://www.xbox.com/kinectMicrosoft.

3 The Point Cloud Library (or PCL) is a large scale, open project for 2D/3D image and point cloud processing.

4
http://www2.imm.dtu.dk/~aam/.
